# The Use of Virtual Reality to Alter Physical Activity by Targeting the Built Environment

**DOI:** 10.1007/s40572-025-00506-6

**Published:** 2025-11-22

**Authors:** Amanda N. Spitzer, Dan J. Graham

**Affiliations:** 1 Psychology Department, Colorado State University, 1876 Campus Delivery, Fort Collins, CO 80523 USA; 2 Department of Community and Behavioral Health, Colorado School of Public Health, Fort Collins, USA

**Keywords:** Built environment, Virtual reality, Physical activity, Walkability, Bikeability

## Abstract

**Purpose of Review:**

Virtual reality (VR) has emerged as a novel approach to research built environmental determinants of physical activity for its ability to address issues of causality, which have historically plagued the discipline. The purpose of this narrative review is to identify the methods by which VR technology has been adapted for use within the research area.

**Recent Findings:**

Current built environmental VR research examining physical activity overwhelmingly targets walking and cycling. Despite spanning few types of PA, we observe diverse VR methodologies and patterns of applications within research areas.

**Summary:**

In this review, we explore how capabilities of current VR technology, specifically simulation development and travel, have shaped research questions, validity, and generalizability. We identify future innovations that may address these limitations. Finally, we encourage future research applying this powerful research tool to investigations of built environmental factors promoting types of physical activity apart from walking and cycling.

**Supplementary Information:**

The online version contains supplementary material available at 10.1007/s40572-025-00506-6.

## Introduction

Physical activity (PA) benefits health in many ways, both short-term and long-term [1, 2]. PA reduces risk of chronic diseases, including cardiovascular disease [3], more than a dozen types of cancer [4], Type 2 diabetes [5], and other chronic illnesses [1]. PA also benefits mental health (e.g., preventing and treating stress, depression, and anxiety [6, 7]). Despite overwhelming evidence of the health benefits of PA, most Americans do not meet PA guidelines [8, 9], making inactivity a public health crisis. For example, Santos and colleagues [10] predict that in the current decade alone, 3.4 million cancer cases will be attributable to physical inactivity if PA levels remain unchanged.

Because built environments affect the behaviors (including the PA [11]) of all individuals who interact with those environments, one promising social ecological approach for reducing illness and preventable death is environmental modification to increase community-level PA [12]. Environments can be made more conducive to PA in ways that make community-level PA safer and more attractive. Many are now calling for changes to built environments to facilitate PA, including groups such as the World Cancer Research Fund/American Institute for Cancer Research and the American Cancer Society [8, 13].

Unfortunately, it is not always clear which environmental changes will increase PA. Indeed, randomly assigning individuals, streets, or communities to experience various environmental changes would be expensive, time-consuming, or even impossible. Thus, most evidence connecting the built environment to PA is correlational [14–17]. The most used method to understand how environments relate to PA is measuring geographic characteristics of locations where people commonly engage in PA [17, 18]. This research identifies associations between built environmental features and PA; such research cannot prove causation (as correlations are subject to third-variable problems and selection biases, e.g., people who are more active may choose neighborhoods with certain PA-friendly features, like walking paths).

Some data are gathered by natural experiments, most commonly through assessment of PA before and after an environment undergoes an intervention [17, 19]. These interventions are regularly multifaceted, comprised of built environmental changes and increased programming; thus, it is not possible to identify the influence of any given modification. Additionally, intervention-based natural experiments carry pitfalls of comparison site selection, delayed construction, and confounding variables, which limit their support for causal inferences [20]. A less common approach of natural experiments measures PA before and after an individual relocates to a new neighborhood [21, 22]. As areas tend to differ on many dimensions, these studies have difficulty determining whether/which environmental difference(s) between areas led to any changes in PA.

True experiments studying environment/PA relationships are rare. Those few that exist have had participants view photographs/videos of various environments (e.g., city streets with vs. without trees) and report their activity intentions/preferences [23, 24]. Similarly, think-aloud protocols ask participants to share their perceptions and preferences either while in the environment or in response to participant-provided photos of places that they find walkable or unwalkable [25–27]. These studies all investigate PA intention or preferences as their primary outcome, which is not ideal as PA intention and PA behavior do not always align [28].

Virtual reality (VR) enables researchers to experimentally assess the effects of environmental modifications on PA in a way that was not previously possible. VR technology immerses users in virtual environments by occluding the real-world environment [29]. Thus, researchers can place participants in modifiable environments, meaning participants can experience an urban area in its current form and with various changes. This technology affords true randomized trials and a high degree of control, thereby enabling causal conclusions regarding which specific interventions are most effective at promoting PA. Randomized VR trials, then, can provide causal information about intervention effectiveness in a similar vein to randomized controlled drug trials, allowing policymakers and planners to test whether interventions are more effective than the status quo in producing desired outcomes (e.g., greater walking and/or cycling in an environment) prior to making changes to the physical environment in question.

The purpose of this review is to identify the methods by which immersive VR technology has been adapted for use within built environmental research addressing PA. As the advent of VR technology has the potential to greatly strengthen causal inferences in this research area, we find it critical to synthesize its current applications and limitations. Through this review, we hope to equip readers with a high-level understanding of VR technology, its present abilities and issues as well as provide future directions that will advance built environmental PA research and practice.

## Definitions

### *Virtual Reality (VR)*

We have limited this review to immersive VR applications in which a virtual world is presented to a user as if it were the physical environment [29]. This regularly includes enveloping the user and occluding the real physical environment. In doing so, we have excluded single or multi-monitor approaches that are less immersive [30]. For more nuance regarding the mixed reality continuum, please refer to Skarbez et al. [31] and Jerald’s seminal book [29].

### *Presence*

Presence is the “feeling of being there” [32]. A central reason for utilizing immersive technologies, like VR, is their ability to induce presence [30].

### *Built Environment*

The built environment is broadly defined as the human-made physical environment [33]. This includes components at various scales, from sidewalk quality to land use.

### *Physical Activity (PA)*

PA is an inclusive term for body movements that expend additional energy beyond the basal metabolic rate [34]. It includes structured exercise as well as unstructured active behaviors, like cleaning and gardening.

## Method

Following non-exhaustive narrative review techniques, we searched Academic Search Ultimate, APA PsychINFO, IEEE Xplore, ProQuest, PubMed, SPORTDiscus, and Web of Science Core Collection for journal articles and conference proceedings using keywords and Boolean operators, such that captured articles were required to have at least one keyword in each of three primary topics: VR (e.g., virtual reality, immers*), built environment (e.g., neighborhood*, physical infrastructure), and PA (e.g., physical activit*, exercis*). For brevity, a list of search terms can be found in Supplemental A. Search terms, truncation, proximity searching, phrase searching and searched fields were tailored to each database. Search results were further restricted to those published in or after 2020. Inclusion criteria were as follows:

1. The publication uses virtual reality as defined above. Thus, publications concerning augmented reality or other forms of mixed reality were excluded.
2. The publication addresses the influence of built environments, as defined above, on perceptions and/or behavior related to PA. Qualities of traffic without a physical infrastructural root (e.g., speed) have been excluded from the present review as they do not meet our definition of built environment.
3. The ultimate objective of the research is to modify real-world built environments, even if that objective is not imminent. Accordingly, publications focused on gait-mechanics in VR and those aimed at adoption of PA in VR (e.g., exergaming) were excluded.
4. The publication must be written in English.

## Key VR Decision Points

Immersive VR encompasses a diverse set of technologies: For any given application, researchers must make innumerable methodological choices that influence the conclusions of their work. Below we briefly describe several key VR decision points and corresponding approaches common in the reviewed research area.

### *VR Equipment Systems*

There are two primary VR equipment systems. CAVE (Cave automatic virtual environment) set-ups project the VR environment onto 3 + walls surrounding the user and, in some models, the floor [30]. Head-mounted displays (HMDs) are comprised of one or more screens that are suspended directly in front of the user’s eyes [29]. HMDs are generally less expensive [29] and induce more presence [35].

### *VR Simulation Development*

Currently, VR research into the built environment’s effects on PA primarily uses one of two VR development approaches, The 360-degree camera development strategy captures an existing environment as either a still photograph or dynamic video. The second development strategy in current research is 3D modeling, in which the geometry of an environment is generated utilizing software such as SketchUp [36]. For reasons that are outside the scope of this review, VR development approaches may guide a researcher’s choice of travel modes in VR. For example, it is more difficult to combine simulations created with 360-degree cameras with the overground/real walking technique [37].

### *VR Travel Techniques*

Another methodological decision VR researchers must make regards how participants will move in VR. Particularly in built environmental research, participants may need to navigate virtual environments that are larger than the available physical space. Below is a non-exhaustive list of travel techniques used in VR research examining built environmental factors of PA. More information on VR travel can be found in Jerald [29] and LaViola et al. [30].

1. A few studies automate movement, such that the user’s perspective moves independent of any input [38–40]. That is, first-person videos of moving through the environment are either recorded or pre-rendered and played for the participant.
2. In other studies, users manipulate a physical device, such as a joystick [41], to move their VR perspective [42, 43].
3. Arm-swinging/walking-in-place, though rarely used [44], moves the user’s perspective when they swing their arms or step-in-place while their arm/leg movements are tracked by VR equipment.
4. Similarly, use of omnidirectional treadmills, which allow users to walk in all directions, are infrequent [45].
5. Overground/real walking moves the user’s perspective as they naturally walk. The user’s distance and rotation in the real world are mapped at a 1:1 ratio to those in VR.

With the exception of automated movement, these VR travel techniques allow users to self-navigate through the virtual environment.

## Current State of Literature

**Fig. 1:**
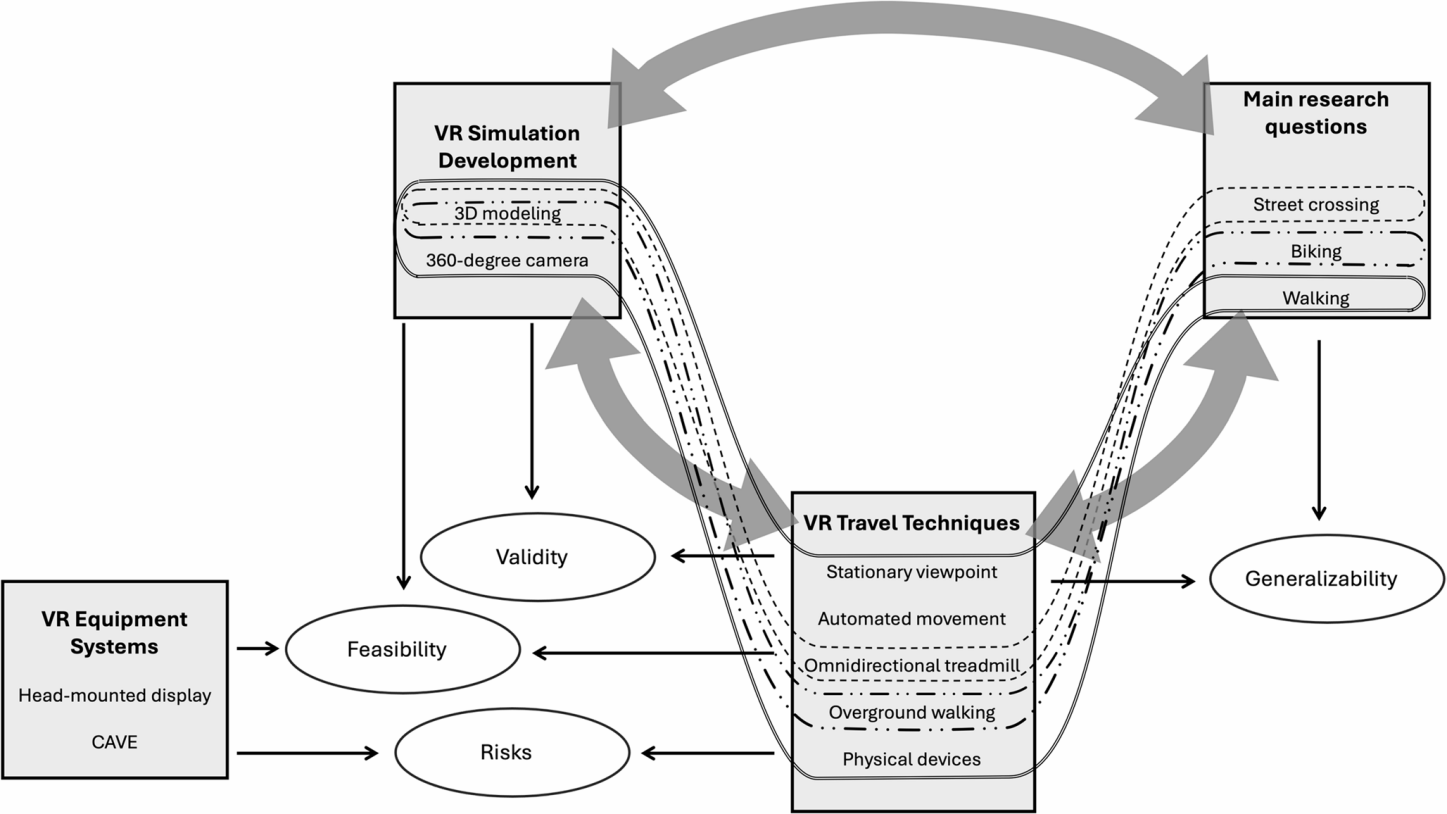
Methodological framework. *Note. *Dashed and double-line regions indicate observed patterns between VR simulation development approaches, VR travel techniques, and research topics

Presently, published research utilizing VR to investigate built environmental factors influencing PA overwhelmingly concerns transportation behavior, namely walkability and bikeability. Table 1 lists recent work in the area and is illustrative of research trends. Based on our findings, we present a framework visualizing patterns between VR methodology, investigated topics, and research issues in Fig. 1.

**Table 1:** Select research using VR to investigate built environmental factors of PA

Targeted PA	Primary outcome(s)	Reference	Study design	*n*	Primary built environmental factor(s)	Equip. system	Dev. approach	Travel mode
Walking	Comfort	[41]	Within	16	Sidewalk width	HMD	3D modeling	Joystick
	General walkability	[45]	Within	4	Greenery	HMD	3D modeling	Omnidirectional treadmill
		[46]	Within	50	Existing street environments	HMD	360 videos	Unknown
		[42]	Mixed	48	Gehl’s guidelines [47]	HMD	3D modeling	Hand controller buttons
		[48]	Within	16 in PA group	Pedestrian priority streets/Shared streets	HMD	3D modeling	Unknown
		[49]	Unknown	35	Existing street environments	Unknown	360 photos	No travel
		[50]	Within	32	Greenery, Sidewalk width	HMD	360 photos	No travel
		[51]	Within	40	Land use, Sidewalk addition, Traffic lanes	HMD	360 photos	No travel
		[52]	Within	40	Street configuration	HMD	360 videos	No travel
		[38]	Mixed	57	Larranaga et al.‘s findings [53]	HMD	3D modeling	Automated movement
	Pedestrian Stress	[44]	Within	35	Greenery, Ground murals	HMD	3D modeling	Arm-swinging/Walking-in-place
	Safety/Security	[54]	Within	50	Pedestrian priority streets/Shared streets	HMD	360 videos	No travel
		[43]	Mixed	102	Sidewalk lanes	HMD	3D modeling	Keyboard
	Street-crossing safety	[55]	Mixed	60	Greenery, Street lighting, Traffic lanes	HMD	3D modeling	No travel
		[56]	Mixed	200	Pedestrian priority streets/Shared streets	HMD	3D modeling	Overground/Real
		[57]	Mixed	200	Crosswalk design, Greenery, Sidewalk addition, Signage, Street parking	HMD	3D modeling	Overground/Real
		[58]	Mixed	48	Traffic lanes	HMD	3D modeling	Overground/Real
		[59]	Within	51	Crosswalk design	HMD	3D modeling	Overground/Real
		[60]	Within	99	Median addition	HMD	3D modeling	Overground/Real
		[61]	Within	38	Crosswalk addition	CAVE	3D modeling	Overground/Real
		[62]	Within	50	Median addition, Traffic lanes	HMD	Unknown	Overground/Real
		[63]	Within	49	Crosswalk design	HMD	3D modeling	Overground/Real
		[64]	Within	43	Pedestrian priority streets/Shared streets, Crosswalk design	HMD	3D modeling	Overground/Real, Joystick
		[65]	Mixed	178	Crosswalk design	HMD	3D modeling	Overground/Real
		[66]	Mixed	171	Median addition	HMD	3D modeling	Overground/Real
Cycling	General bikeability	[67]	Unknown	78 in PA group	Traffic lanes	HMD	360 videos	No travel
		[68]	Mixed	150	Bicycle lane design	HMD	3D modeling	Stationary bike
		[69]	Mixed	70	Cleanliness, Greenery, Intersection removal, Land use, Protected bike paths	HMD	3D modeling	Stationary bike
		[70]	Within	126	Existing street environments	HMD	3D modeling	Unknown
	Visual attention	[71]	Unknown	22	Existing routes	HMD	360 videos	Unknown
	Safety/Security	[72]	Within	46	Intersection design	CAVE	3D modeling	Stationary bike
		[73]	Within	50	Bicycle lane design	HMD	3D modeling	Stationary bike
		[74]	Within	50	Bicycle lane design	HMD	3D modeling	Stationary bike
		[75]	Mixed	208	Cleanliness, Street lighting, Visibility obstructions (incl. greenery)	HMD	3D modeling	Stationary bike
		[76]	Mixed	52	Cleanliness, Street lighting, Visibility obstructions (incl. greenery)	HMD	3D modeling	Stationary bike
		[43]	Mixed	102	Sidewalk lanes	HMD	3D modeling	Stationary bike
General PA	Affect	[77]	Within	48	Fitness facilities, Greenery	HMD	3D modeling	Unknown

Equip.: equipment. Dev: development. Within: within-subjects design. Mixed: mixed design

### Walking Research

Currently within built environmental PA research, VR technology has most commonly been used to study walkability [38, 41, 42, 44–46, 48–52, 78, 79], with a subset of research focusing on pedestrian street-crossing [55–66]. Within general walking research, studies immerse participants in multiple built environmental conditions. Depending on study questions, these conditions may reflect a single environment with select modifications [38, 41–45, 48] or entirely different environments [39, 46, 49, 51, 52, 54, 80]. Nearly all walking studies measure at least one self-reported perception of the environment [38, 41, 42, 44–46, 48–52, 54, 78], commonly safety [48, 50, 52, 54, 78]. As described below, the utility of behavioral measures is influenced by VR methodological decisions.

Most studies that expose participants to multiple different environments that have not been manipulated use simulations developed by the 360-degree camera development strategy [39, 46, 49, 51, 52, 54, 67, 71, 80], indicating a bidirectional relationship between the selected VR development approaches and available study questions. In fact, the 360-degree camera approach is more frequently used to study general walkability [39, 46, 49–52, 54] than pedestrian street-crossing (0) or cycling [67, 71]. Additionally, many walking studies that immerse participants in different environments created via 360-degree camera have participants stay stationary [49–52, 54]. This limits the types of measurement that are available for this research: Certain behavioral indicators, such as walking intensity, are not appropriate. Instead, these studies primarily collect self-reported data from discrete perspectives [49–52, 54].

On the other hand, 3D modeled simulations are most commonly applied to study specific manipulated environmental factors of walking [38, 42, 45, 48, 78, 79]. For example, participants have been repeatedly immersed in an environment with varied sidewalk width [38, 41, 50]. The VR travel modes adopted in research utilizing 3D modeled simulations to examine specific manipulations are quite diverse and span most discussed techniques (Automated movement [38], physical device [41, 42, 48], arm-swinging [44], omnidirectional treadmill [45]). Overground/real walking can be difficult to implement as these studies assess large areas (i.e., urban blocks) that cannot be mapped 1:1 onto a research space. Again, certain measurements can be difficult to interpret with walking research designs in which participants do not overground walk. For example, the speed at which participants move through a virtual environment by physical device [42] and arm-swinging [44] has been recorded as a proxy of walking speed and interpreted as a measure of enjoyment [42] and stress [44]. There are only several studies that utilize overground/real walking at an urban scale. They collect walking duration data [81, 82], but these protocols must use “spotters” to redirect participants when they near a physical boundary [29]. For a systematic review of walkability research utilizing VR, see Ghanbari et al. [83]. Additionally, recent VR literature examining walkability has included several feasibility/pilot studies [45, 78, 79] and future study protocols [84–86], indicating the emerging state of this field.

Comparatively, research into road crossing safety for pedestrians using VR methodology [55–66] is a more homogeneous landscape. Much pedestrian-focused road-crossing research [56–60, 63–66] adopts a study procedure wherein participants complete multiple trials comprised of crossing the same street with various environmental interventions introduced, such as crosswalk design [57, 64, 65]. Aligned with other research using a single manipulated environment [38, 42, 45, 48, 78, 79], these street-crossing studies show a strong preference for the 3D-modeling development approach [55–61, 63–66, 87]. Relatedly, overground/real walking is the de facto travel mode for street-crossing studies [56–63, 65, 66, 85]. Discussed further within the feasibility section, this VR travel mode is enabled by the scale of the research question. Moreover, the intersection-scale walk in many street-crossing protocols fits within a research space [56–63, 65, 66, 85], so alternative VR travel modes that navigate large virtual spaces within smaller physical spaces are unnecessary. Since participants are naturally walking in VR, these studies commonly measure real walking speed [56–58, 63, 65, 87]. There is a recent trend to examine how built environmental factors influence road-crossing safety in an anticipated future of autonomous vehicles [61], particularly regarding the addition of medians [60, 62, 66]. For a systematic review of pedestrian and cycling safety research utilizing VR, refer to Sudhakaran et al. [88].

### Cycling Research

Like walkability research, bikeability research is developing and features recent pilot studies [71, 79, 89, 90]. Ghanbari et al. [83] provides a systematic review. Recent built environmental cycling studies using VR address questions spanning general bikeability [67–70, 79, 89, 90] to cycling safety [43, 72–76]. Within this research, immersing participants in fully separate environments [67, 70, 71] (e.g., different cycling routes [71]) is less prevalent than repeatedly placing them in an environments with specific manipulated factors [43, 68, 69, 72–74] (e.g., added greenery [69]). Only a few studies combine these approaches [75, 76]. Despite the split in research designs, this research area demonstrates a clear pattern of VR methodological decisions. Most studies utilize a 3D-modeling approach [43, 68–70, 72–76, 79, 89, 90]. Notably, the investigated PA itself uses a physical device for locomotion (i.e., the bicycle); thus, there is a clear VR equivalent: the stationary bike [43, 68, 69, 72–76, 79, 89, 90], which can be programmed to provide speed and/or direction data to move the user’s VR perspective. Because using a real-world cycle and a stationary cycle are quite analogous, studies in this area are able to collect PA intensity data with validity (e.g., speed [73], heart rate [73, 74]). Nevertheless, built environmental cycling research using VR has relied heavily on self-reported outcomes [43, 67–70, 72, 75, 89, 90].

### Gaps

Due to the overwhelming preference in the literature for HMDs [38, 41–46, 48, 50–52, 54–60, 62–64, 66–71, 73–79, 90], we did not observe patterns regarding equipment systems. Still, HMDs differ in their capabilities affording some researchers to collect eye-tracking data while participants walk [44, 45, 51, 79] or cycle [71, 79]. These data are most commonly used to identify salient features of environments [44, 45, 51, 59, 71, 73] and quantify cognitive load [44, 59, 73, 79].

There seems to be a gap within current literature using VR to assess built environmental interventions promoting PA, namely studies targeting PA forms aside from walking and cycling. We were able to identify one recent study concerning fitness facilities [77]. Participants in this study experienced courtyards with or without greenery and fitness facilities in VR while electrodermal activity (EDA) data was collected as a measure of affective state.

### Assumptions

The primary assumption of research using VR to examine a real-world phenomenon is that through the elicitation of presence, VR can induce realistic responses. The validity of this assumption has been examined, and the research presented in this review should be interpreted in context with the current generation of VR technologies’ ability to elicit realistic transport-related responses as compared to both the real world and to conventional visualization techniques.

Regarding the similarity of VR and real-world responses, both Nishio and Ito [91] and Spitzer et al. [82] report matching perceptions of pleasantness and relaxed mood for a VR environment and its real-world counterpart. Moreover, VR and real-world conditions did not differ regarding participants’ motivation to walk [82, 91]. These studies also observe differences between VR and real-world judgements, including those of spaciousness. Angulo et al. [87] indicate that pedestrians cross the street in similarly-sized gaps between cars in VR as in the real world though they wait longer before crossing in VR. However, Schneider et al. [92] have observed riskier street-crossing in VR than in the real world. Spitzer et al. [82] and Spitzer et al. [81] compare walking behavior in a real-world environment with that in an equivalent VR environment. Their results indicate a complex relationship between the completion order of VR and real-world conditions and the similarity of walking duration – potentially pointing to a novelty effect of VR, wherein participants are more interested in a novel VR walking experience than in the corresponding familiar real-world experience.

Indicating marginal benefit over 2D media, research comparing self-reported perceptions of environments experienced in VR with those based on 2D videos [93] and images [80] has concluded that both approaches are acceptable, and that VR can outperform the 2D medium in several perceptual domains, including environment attractiveness and safety [38]. Kim and Lee [39] found that walkability assessments performed by trained auditors based on VR visualization largely matched real-world assessments better than those based on 2D images like Google Street View. Concerning publics unfamiliar with urban design, Meenar and Kitson [40] determined that focus groups involved in participatory design were more engaged and understood the urban designs better when VR was used as design visualization compared to 2D images.

In sum, we do not blindly accept the assumption and argue that VR is the same as the real world; indeed, we are aligned with Newman and colleagues when they say,

*“There is no contention that*,* currently*,* surrogate [environmental] experiences can or should replace the real thing … However*,* this has not invalidated their use within research*,* as surrogate experiences can enable access to environments when physical presence is not possible. Additionally*,* computer generated environments can be customised to match research needs; thus*,* permitting new lines of enquiry within the field”* [94].

## Current Issues in the Field

### Generalizability and Validity

Joseph et al. [36] differentiates the interrelated concepts of visual realism (i.e., looking real) and behavioral realism (i.e., eliciting realistic responses). They state that VR realism is resource-intensive, and researchers should consider the importance of each type of realism for their specific goals. These considerations may aid researchers in selecting a VR development approach. For example, the 360-degree camera approach results in higher visual realism than the alternative [95]. Behavioral realism is of particular importance to generalizability to real-world planning decisions. Notably, VR environments with low visual realism can still have adequate behavioral realism [94]. In addition to visual stimuli, many researchers incorporate sounds into their simulations [39, 40, 48, 52, 55, 56, 60, 63, 65, 67, 72, 81, 82, 87, 90, 92]. Still, environmental stimuli that are not audiovisual influence PA [12]. There have been attempts to develop VR systems that add stimuli that are not audiovisual [40] (see Neo et al. [96] for a review); however, they have not been widely adopted within PA research.

As previously mentioned, the selection of a VR travel mode can restrict or enhance the researcher’s ability to measure certain variables, particularly behavioral variables, with validity. Jerald [29] notes that when using overground walking, users must be motivated as they are required to expend energy to locomote. In PA research, this energy expenditure may increase the behavioral realism of the study [36] depending on the research questions. Furthermore, VR travel modes may affect participants’ performance on unrelated study tasks completed in VR [30]. Arduous locomotion can distract users. For example, using the current generation of omnidirectional treadmills can be onerous and dissimilar from overground walking [97]. In sum, VR guidelines encourage developers to select a VR travel technique that fits their objectives [30].

Lastly, researchers’ decisions regarding whether to immerse participants in environments that are identical apart from manipulated environmental elements or environments that differ in many ways can affect the studies’ applicability to practice. When entirely different environments are compared, researchers struggle to determine which environmental factors caused responses. On the other hand, environments with manipulated elements may not reflect real-world variation. Based on our review, there are only a few publications that take a hybrid approach, where multiple environments have elements manipulated [50, 75, 76].

### Feasibility

VR studies are resource-intensive. VR development, particularly 3D modeling approaches, can be complex and lengthy. This process can be laborious and require specialized skills; thus, several researchers have hired expert firms to create 3D-modeled VR environments [65, 66, 69]. Researcher decisions regarding VR equipment systems and VR travel modes can influence space needs. For example, certain HMDs are standalone [36], allowing data collection to be mobile and occur in rooms without a VR-enabled computer. Use of overground walking can require a large physical space [81]. Research personnel must be highly trained to work with VR devices, particularly when unexpected simulation errors occur [45]. Concerning time spent with participants, a portion of the study session should be dedicated to acclimating them to VR [36]. In short, VR data collection requires extensive training and time. Possibly due to these resource-demands, many studies using VR methodology investigating the effects of environments use modest sample sizes [83]. Smaller sample sizes result in less precise statistical estimates, thereby reducing the studies’ power to detect built environmental effects using null hypothesis significance testing [98]. Essentially, the robustness of a literature based on small sample sizes is low [99].

### Risks

The primary risk to individuals participating in VR research is experiencing symptoms akin to motion sickness, which have been named “cybersickness” [100]. Common symptoms include nausea and disorientation. Cybersickness is noted as a product of incompatible vestibular (i.e., movement and balance) and visual stimuli [30]. Susceptibility to cybersickness is affected by individual factors (e.g., age [101]), simulation factors (e.g., frame rate [101]), and hardware factors (e.g., HMD model [100]). Furthermore, researcher decisions regarding VR travel affect risk of cybersickness [100, 102] as these symptoms may be experienced when the user’s movement input does not match the movement of their perspective in VR [29]. Accordingly, automated movement has a high likelihood of inducing cybersickness [29], and overground/real walking has a relatively low likelihood of inducing cybersickness [29, 102].

## Future Directions

As reviewed here, VR research to alter PA by targeting the built environment is an emerging research area that has grown substantially in the past 5 years. We anticipate future research will do an ever-improving job of replicating real-world conditions within VR. For example, the ability to generalize VR results to the real world will benefit from multimodal approaches, in which multiple users can simultaneously locomote by different means of transport, such as research by Sim and Cho [43]. In their study, participants concurrently moved in a single simulation via stationary bike or scooter, or as a pedestrian (using the physical device approach to VR travel). Further, these multiplayer VR games will better reflect real-world conditions and enhance the policy relevance of VR data. Of course, design decisions made by transportation planners affect all road users. Most current VR transportation research is limited by its consideration of one transport modality, thereby offering decisionmakers insight restricted to one type of stakeholder. Multimodal approaches will grant decisionmakers findings about many road users and their interactions. An additional future direction that we anticipate is the establishment of collaborations between researchers and policymakers, in which VR research is used to predict behavior changes in response to environmental modifications under consideration for the real-world. Using VR in this manner - as a pre-testing tool - has two primary benefits. First, it ensures that findings are directly applicable to policymakers by addressing environmental factors of interest to them and doing so in the local context. Second, it provides an opportunity to compare predictions made using VR with subsequent real-world data to better understand when and how VR accurately simulates PA behavior.

We also anticipate that future research will continue to investigate new approaches to locomotion. Current approaches suffer from various limitations, as detailed above, and it is likely that many of these limitations can be addressed through improvements to existing technologies (e.g., omnidirectional treadmills). Redirected walking is also a promising technique: It is similar to overground/real walking as it moves the user’s perspective while they naturally walk [103]. However, redirected walking manipulates the mapping ratio to enable large VR spaces to be navigated within smaller physical spaces. For example, the user’s perspective can be adjusted to induce the user to physically walk along a curved path although their trajectory in the VR simulation is a straight line. A limitation of this approach is that a relatively large physical space may still be required. We have not identified a study in the current research area that utilizes redirected walking, but we anticipate that this approach will be used to great effect in the future. Improving VR travel techniques will greatly augment this research area’s generalizability through improving the behavioral realism of transportation in VR. Furthermore, reductions in physical space requirements will boost the feasibility of data collection while declines in cybersickness will lessen risks. Lastly, it will increase measurement validity and grant opportunities to address new walkability research questions by minimizing barriers to measuring behavioral variables of walking.

Finally, although this review found the majority of relevant studies focused on walking and cycling, it is likely that future research would benefit from exploring non-transportation PA and even non-exercise PA. There are many types of PA [104], and changes to built environments can facilitate or impede their conduct. Expanding VR research into these effects beyond walking and cycling could provide a more holistic view of PA and the many contexts in which it occurs, including non-traditional settings that might better reflect population subgroups not well represented in the current research. We encourage researchers to innovate designs that harness VR to study the effects of such environmental changes as park renovations on PA behaviors other than walking and cycling (e.g., the effects of new or improved sports fields/facilities on playing ball sports). Similarly, some neighborhood modifications establish community gardens that may encourage residents to engage in gardening-associated PA. VR research may help provide data regarding the PA effects of changes to these assorted environments. With VR studies into diverse built environmental factors, this research area may contribute causal evidence to policymakers for broad applications.

## Conclusions

Researchers have begun using VR to alter PA by targeting the built environment. Although VR behavior is not identical to real-world behavior, the emerging VR literature suggests that VR can capture realistic affect, cognition, and behavior in many PA-relevant domains. Based on the dozens of studies described here, VR has potential to reshape causal inferences in the field of PA promotion via environmental modification. Nonetheless, it is important to understand that VR is still emerging. Current limitations generate challenges, namely feasibility of data collection and heterogeneity regarding generalizability and measurement validity. Through this review, we have identified patterns of VR applications and methodological decisions. We have explored how these decisions influence generalizability, validity, feasibility, and risks. In doing so, we have observed gaps and highlighted select technological advancements that can address the current limitations of this research approach. We are sure we have only identified a small fraction of the ways that researchers will use this technology to advance the discipline. We look forward to the growth of this field and the emergence of novel solutions to current problems as well as inevitable innovations we have not anticipated.

## Key References

- Birenboim, A.; Ben-Nun Bloom, P.; Levit, H.; Omer, I. The Study of Walking, Walkability and Wellbeing in Immersive Virtual Environments. *Int. J. Environ. Res. Public. Health* **2021**, *18*, 364, doi:10.3390/ijerph18020364.
  - A pilot study for a VR procedure assessing walkability that provides guidelines for similar research.
- Facchini, G.; Larranaga, A.M.; Cândido Dos Santos, F.A.; Dos Santos, M.L.; Nodari, C.T.; Presta García, D.S. Virtual Reality in Stated Preference Survey for Walkability Assessment. *Transp. Res. Part Transp. Environ.* **2025**, *139*, 104545, doi:10.1016/j.trd.2024.104545.
  - The publication concludes that walkability judgements of VR street environments are an improvement over those of 2D visualization techniques.
- Ghanbari, M.; Dijst, M.; McCall, R.; Perchoux, C. The Use of Virtual Reality (VR) to Assess the Impact of Geographical Environments on Walking and Cycling: A Systematic Literature Review. *Int. J. Health Geogr.* **2024**, *23*, 15, doi:10.1186/s12942-024-00375-6.
  - A systematic review specific to walkability and bikeability research utilizing VR.
- Joseph, A.; Browning, M.H.E.M.; Jiang, S. Using Immersive Virtual Environments (IVEs) to Conduct Environmental Design Research: A Primer and Decision Framework. *HERD Health Environ. Res. Des. J.* **2020**, *13*, 11–25, doi:10.1177/1937586720924787.
  - An overview of the application of VR in environmental research with an emphasis on types of simulation realism and their relative costs.
- Neo, J.R.J.; Won, A.S.; Shepley, M.M. Designing Immersive Virtual Environments for Human Behavior Research. *Front. Virtual Real.* **2021**, *2*, 603750, doi:10.3389/frvir.2021.603750.
  - A systematic review of VR applications in research conducted to identify factors of VR simulations critical to study success.

## Supplementary Information

Below is the link to the electronic supplementary material.

Supplementary Material 1

## Data Availability

No datasets were generated or analysed during the current study.
