# Supplementary material for: The Use of Virtual Reality to Alter Physical Activity by Targeting the Built Environment
Source: Curr Environ Health Rep. 2025 Nov 22;12(1):46. doi: 10.1007/s40572-025-00506-6 (PMC12640343; doi:10.1007/s40572-025-00506-6)
Supplement: Supplementary file 1 — Supplementary Material 1 [file 40572_2025_506_MOESM1_ESM.docx]

**Supplemental A: Search Terms**

VR search terms: CAVE OR “head mounted display” OR “head-mounted display” OR HMD OR immers* OR simulated OR simulation OR simulation VR OR “virtual environment” OR “virtual environments” OR “virtual environment*” OR “virtual reality” OR “virtual simulation” OR VR OR 3D-CAVE OR “360 degree environment” OR “360 degree environments” OR “360 degree video” OR “360 degree videos” OR “360 degree environment” OR “360 environments” OR “360 video” OR “360 videos” OR “360° environment” OR “360° environments” OR “360° video” OR “360° videos” OR (360* N2 environment) OR (360* N2 video)

AND

Built environment search terms: “bike lane” OR “bike lanes” OR “bike path” OR “bike paths” OR bikeab* OR “built environment*” OR “calisthenics park” OR “calisthenics parks” OR (calisthenics N3 parks) OR (calisthenics N3 park*) OR “city infrastructure” OR (city N2 infrastructure) OR “city planning” OR “cycle lane” OR “cycle lanes” OR “cycle path” OR “cycle paths” OR “cycle route” OR “cycle routes” OR “cycle trail” OR “cycle trails” OR cycleway OR “cycling infrastructure” or (cycling N2 infrastructure) OR “environment design” OR “fitness course” OR “fitness courses” OR (fitness N3 course) OR (fitness N3 courses) OR (fitness N3 course*) OR “fitness facility” OR (fitness N3 facility) OR “fitness facilities” OR (fitness N3 facilities) OR (fitness N3 facilit*) OR “fitness park” OR “fitness parks” OR (fitness N3 park) OR (fitness N3 parks) OR (fitness N3 park*) OR “fitness station” OR “fitness stations” OR (fitness N3 station) OR (fitness N3 stations) OR (fitness N3 station*) OR (fitness N3 stations) OR foothpath OR infrastructure OR “natural environment” OR “natural environment*” OR neighborhood OR neighborhood* OR neighbourhood OR neighbourhood* OR “open space” OR “outdoor environment” OR “outdoor environment*” OR park OR “pedestrian environment” OR “pedestrian environment*” OR “pedestrian infrastructure” OR (pedestrian N2 infrastructure) OR “pedestrian pathway” OR “pedestrian trail” OR “pedestrian trails” OR “physical environment*” OR “physical infrastructure” OR (physical N2 infrastructure) OR “public place” OR “public places” OR (roadway N3 design) OR sidewalk OR sidewalk* OR “street environment” OR “street environment*” OR “street infrastructure” OR (street N2 infrastructure) OR streetscape*OR “transport infrastructure” OR (transport N2 infrastructure) OR “transportation infrastructure” OR (transportation N2 infrastructure) OR urban* OR “walk path” OR “walk paths” OR “walk trail” OR “walk trails”

AND

PA search terms: “active behavior” OR “active behaviour” OR “active transport*” OR bicycle* OR cycl* OR cycli*OR cycling OR cyclist OR cyclist* OR exercis* OR pedestrian OR pedestrian* OR “physical activit*” OR walk*

*Note.* * refers to truncation. “” refers to phrase searching. N# refers to proximity searching in which # represents the maximum distance between the search terms.
